# Long term control of a maxillary sinus mucoepidermoid carcinoma with low dose radiation therapy: a case report

**DOI:** 10.1186/1748-717X-8-251

**Published:** 2013-10-29

**Authors:** Horia Vulpe, Meredith Giuliani, David Goldstein, Bayardo Perez-Ordonez, Laura A Dawson, Andrew Hope

**Affiliations:** 1 Department of Radiation Oncology, Princess Margaret Cancer Centre, University Health Network, University of Toronto, Toronto, Ontario, Canada; 2 Department of Pathology, University Health Network, University of Toronto, Toronto, Ontario, Canada; 3 Department of Otolaryngology – Head and Neck Surgery, University of Toronto, Toronto, Ontario, Canada

**Keywords:** Mucoepidermoid carcinoma, Radiotherapy, Head and neck, Palliation

## Abstract

Mucoepidermoid carcinoma of the maxillary sinus is a rare malignancy of the head and neck. The location of this tumour near vital structures and its large size at presentation makes surgical resection with negative margins challenging. In incurable cases, relief from symptoms such as epistaxis may be achieved with radiation therapy. We present a case of mucoepidermoid carcinoma of the maxillary sinus that was effectively palliated with a short course of radiation therapy, achieving complete cessation of bleeding, decrease in tumour size, and long term control. We surveyed the literature on mucoepidermoid carcinomas and propose that some tumours may be particularly radiosensitive, benefiting from even short courses of radiation therapy.

## Background

Paranasal sinus malignancies account for 3-5% of all head and neck cancers [1,2]. The vast majority are squamous cell carcinomas; non-squamous histologies are rare. In a retrospective review of 220 nasal and paranasal sinus carcinomas, the most frequent histologic type was squamous cell carcinoma (126 patients), followed by adenoid cystic carcinoma (35), undifferentiated carcinoma (30) and adenocarcinoma (25); only four were mucoepidermoid carcinomas (MEC) [1].

Histologically, MECs include at least three cell types: epidermoid cells, mucous cells and intermediate cells [3,4]. MECs usually originate in the major salivary glands of the head and neck but may also develop in any of the 500–1000 minor salivary glands lining the upper aerodigestive tract, including the oral cavity, pharynx, larynx, nasal cavity and paranasal sinuses [5]. MECs are reported within paranasal sinuses [1,4,6-8], but they remain extremely rare [2-4], and are often grouped with non-squamous carcinomas of different histologic types, grades and sites. This makes it difficult to summarize the characteristics, epidemiology and behaviour of paranasal sinus MECs as a distinct entity.

In general, paranasal sinus malignancies of all histologies are more common in males (1.6-2.0:1; M:F) and the median age of diagnosis is between 50–70 years of age [1,2]. These tumours usually present with advanced stage because expansion within the air-filled sinuses typically causes nonspecific symptoms such as pain, a palpable mass, congestion, swelling, dysphagia and epistaxis [1,2,7,9,10]. All histologic types combined, malignancies of the paranasal sinuses present with locally advanced T3/4 disease in 70-88% of cases [1,2]. Data on the distinct presentation of MECs is sparse because they are often pooled with other salivary gland tumours. There is nevertheless some evidence that they are diagnosed equally late. Bhattacharyya extracted 15 cases of maxillary sinus MEC from the Surveillance, Epidemiology, and End Results (SEER) database and found that 80% presented at advanced tumour stage [11]. Dulguerov et al. report that glandular carcinomas of the maxillary sinus (35 adenoid cystic and four MECs) presented with T3/4 disease 70.3% of the time [1].

Once diagnosed, the large size of the tumour puts it in proximity of vital structures such as the eye, optic nerve, cavernous sinuses and brain. Achieving negative surgical margins may be technically difficult and, in incurable cases, relief from symptoms may be achieved with palliative external radiation therapy. We report here on a case of maxillary sinus MEC which displayed impressive radiosensitivity to low, palliative doses of radiation therapy.

## Case presentation

An 84 year old lady was referred to a local otolaryngologist-head and neck surgeon for a history of epistaxis, occasional hemoptysis, difficulty swallowing, and a fetid odor. She denied pain or significant distress related to these symptoms. She did not drink, smoke or have any known family history of cancer.

Her medical history included resection of a maxillary sinus mass 45 years prior, in a different country. The patient was unaware of the diagnosis and her previous medical records could not be obtained. The prior surgery left her with a surgical defect in the hard palate.

On presentation, her physical exam revealed numbness in the left V2 distribution as well as left sided proptosis. Extra-ocular movements were normal and palpation of the neck did not reveal any lymphadenopathy. Oral and endoscopic examinations showed a surgical defect in the left hard palate extending into the maxillary sinus and a mass visible in the left nasal cavity and maxillary sinus. The remainder of the upper aerodigestive tract was unremarkable.

**Figure 1:**
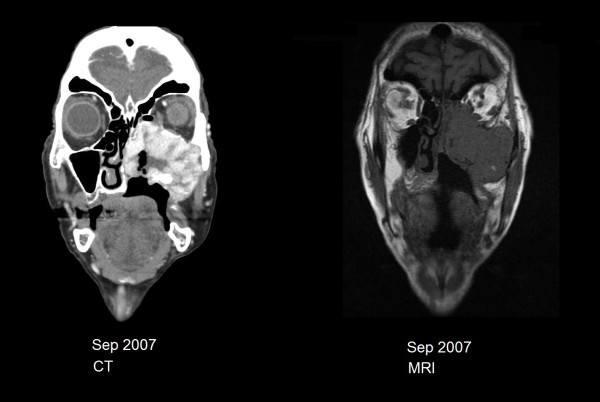
Coronal head and neck computed tomography (left) and magnetic resonance imaging (right) showing tumour size at presentation.

Computed tomography of the head and neck revealed a large heterogeneous and vascular mass at the center of the left maxillary sinus with destruction of the medial, inferior and lateral walls; destruction of the left side of the hard palate; infiltration of the pterygopalatine fissure and infratemporal fossa as well as extension into the nasal cavity. The tumour extended into the left ethmoid air cells, breaching the medial wall of the orbit but remained extraconal, without intracranial extension (Figure 1). MRI further demonstrated mass effect on the ocular musculature causing proptosis and bilateral prominent retropharyngeal nodes suspicious for nodal metastases. The maximum diameter of the mass was 6.5 cm. There was no evidence of distant metastasis on systemic staging.

**Figure 2:**
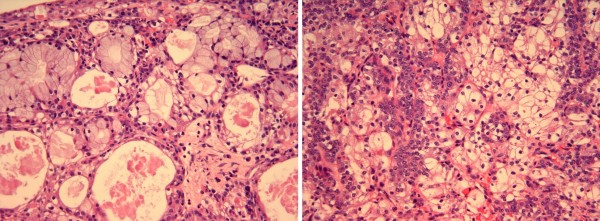
**Left: Intermediate grade mucoepidermoid carcinoma showing small cysts lined by numerous mucous cells (H&E stain, 200X).** Right: Intermediate grade mucoepidermoid carcinoma composed of large numbers of polygonal clear cells and smaller intermediate cells with round nuclei and small nucleoli (H&E stain and, 200X).

Pathologic examination of the biopsy specimen demonstrated small cysts lined by numerous mucous cells, large numbers of polygonal clear cells and smaller intermediate cells with round nuclei and small nucleoli (Figure 2). These findings were consistent with an intermediate grade (II/III) mucoepidermoid carcinoma.

The patient’s case was presented at multidisciplinary rounds and based on the extent of disease, patient performance status and the morbidity related to surgical resection the consensus was not to recommend curative surgery. Rather, the recommendation was for palliative endoscopic debulking to improve proptosis as well as palliative radiation therapy either as an alternative or in conjunction with endoscopic debulking. The patient declined initial treatment but then opted for radiotherapy alone 18 months later when bleeding from the tumour became unmanageable.

**Figure 3:**
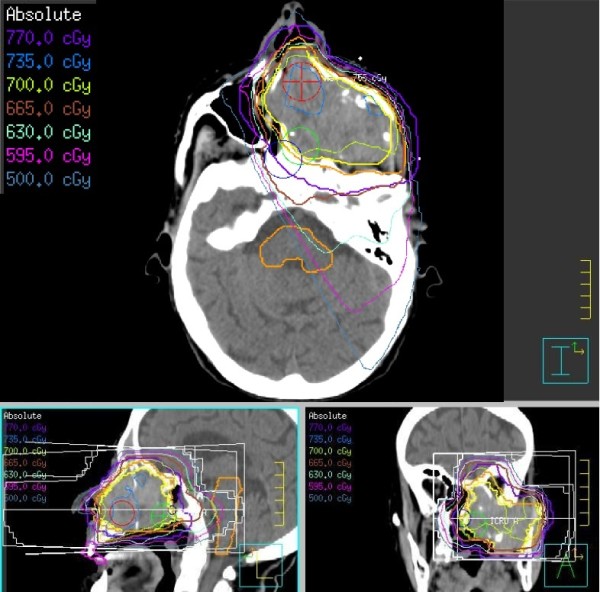
**First radiation treatment plan showing isodose distribution on axial, coronal and sagittal images.** The patient received 700 cGy to the gross tumour volume (yellow isodose line) using right anterior oblique and left posterior oblique conformal beam arrangement.

She received 700 cGy to the tumour using a right anterior oblique and left posterior oblique conformal beam arrangement (Figure 3). Within one week she achieved complete cessation of bleeding, decrease in bulk of her tumour and improved eating. She continued to have some discomfort from the lesion and underwent a second fraction of 700 cGy seven days later. This second fraction used a left lateral, right lateral and right anterior oblique beam arrangement to limit dose the brainstem and optic chiasm. The lenses were largely avoided to prevent morbidity from pain and dry eyes. Estimated cumulative maximal doses to organs at risk were 909 cGy to the brainstem, 1073 cGy to the optic chiasm, 795 cGy to the left eye, 1141 cGy to the right eye, 266 cGy to the left lens and 379 cGy to the right lens. Bleeding did not recur and her capacity to swallow improved as the mass shrunk in size. Given the excellent palliative result, a third planned fraction was not delivered. Her only side-effect from radiation was mild mucositis, which resolved within two weeks.

**Figure 4:**
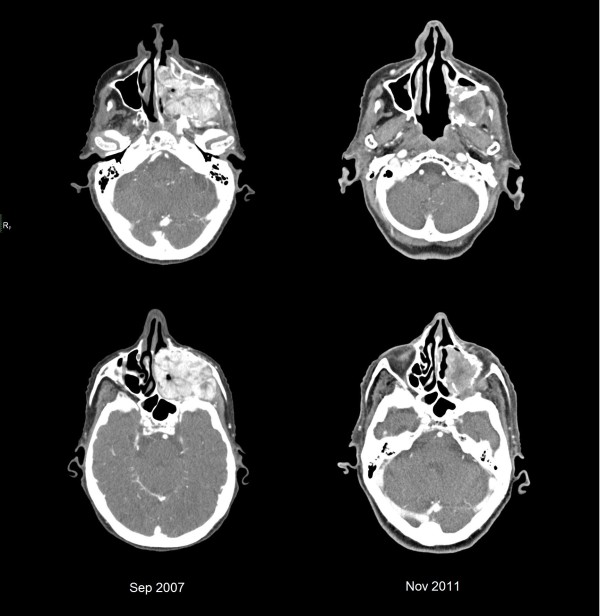
Sequential computed tomography between September 2007 and November 2011 showing decrease in tumour size after a short course of radiation therapy delivered in June 2009.

Follow-up CT two years later revealed a tumour clearly smaller in size measuring 4.5 cm. It abutted the floor of the left orbit, which had been previously displaced and infiltrated by tumour (Figure 4). There were no suspicious lymph nodes. At last follow-up, a little more than four years following completion of radiation therapy, the patient did not have any evidence of progression of her tumour. She continued to have eating difficulties due to the palatal defect which no longer was filled with tumour, but declined to use an obturator. Gastrostomy tube insertion was attempted but subsequently removed at the patient’s request. She maintains caloric intake with liquid nutritional supplements.

## Discussion

Loco-regional control remains of crucial importance in incurable cases of head and neck cancer. Tumour extension into vital structures such as the oral cavity, airway and orbit can be extremely morbid causing impairment in basic human functions and significant disfigurement. Palliative radiation therapy (RT) aims to improve symptoms related to locoregional tumour spread while potentially slowing or halting tumour progression. Because both RT and the patient’s malignancy may cause debilitating side-effects, it can be difficult to find balance between improving quality of life and causing further symptoms such as pain, mucositis and dysphagia. For this reason palliative radiation regimens for head and neck cancer are individualized to each patient based on factors such as performance status and life expectancy [10].

A variety of radiation schedules exist but optimal dose and fractionation scheme remain to be elucidated. Although several trials and reviews have studied palliative radiation therapy regimens in head and neck cancers [9,12-14], these usually contain few, if any, MECs. Chen et al. analyzed 60 patients with SCC of the head and neck treated with various palliative RT regimens and found that the RTOG 85–02 schedule (4440 cGy using 370 cGy fractionation, administered twice a day for 2 consecutive days at 2- to 3-week intervals for 3 total cycles) was associated with the least toxicity, while being equally effective.

Whether this also applies to MECs is unclear and there is generally little consensus on the radiosensitivity of this tumour [15-17]. There is emerging evidence that MECs may exhibit better response to radiation therapy than previously thought in both the curative and palliative setting. A retrospective review from the Princess Margaret Hospital analyzed patients treated with palliative RT to the head and neck and found that patients with non-squamous histologies (including five MECs) had increased overall survival (OS) as compared to SCC (HR 0.52 CI: 0.28–0.96) [10]. In a large retrospective review of 220 patients with nasal and paranasal sinus carcinomas, 5-year carcinoma specific actuarial survival was 79% for patients with glandular carcinoma (mucoepidermoid and adenoid cystic) versus 60% for those with squamous cell carcinoma [1]. A Japanese case report describes a MEC of the larynx that completely responded to curative doses of RT [18].

To our knowledge this is the first case report of a MEC treated with low, palliative doses of radiotherapy, resulting in partial response and long term control for more than four years. This report contributes to the small amount of existing evidence that MECs may be particularly radiosensitive. Consideration should be given to short courses of palliative RT in the management of patients with locally advanced disease who are not suitable for curative treatment, as even low doses may lead to palliation of symptoms and sustained tumor control.

## Consent

Written informed consent was obtained from the patient for publication of this Case report and any accompanying images. A copy of the written consent is available for review by the Editor-in-Chief of this journal.

## Competing interests

The authors declare that they have no competing interests.

## Authors’ contributions

HV researched and drafted the manuscript. LD was the primary radiation oncologist involved in this case. LD and AH substantially contributed to drafting and revision of the manuscript. DG was the primary ENT surgeon involved in the management of this case. MG critically reviewed the manuscript and assumed the patient’s care during follow-up visits. BPO was the primary pathologist involved in this case and contributed to the pathology section of the manuscript. All authors read and approved the final manuscript.
